# Support Needs Among Older Tenants Living in Public Housing in Sweden: Perspectives of Janitors and Maintenance Staff

**DOI:** 10.1177/07334648231169130

**Published:** 2023-04-25

**Authors:** Agata Yadav, Marianne Kylberg, Marianne Granbom, Agneta Malmgren Fänge, Susanne Iwarsson

**Affiliations:** 1 Department of Health Sciences, Faculty of Medicine, 5193 Lund University, Lund, Sweden

**Keywords:** municipal housing company, corporate social responsibility, aging in place, older adults

## Abstract

Neighborhood support can improve aging in place for older adults, but research on the role of public housing staff in supporting older tenants is lacking. Twenty-nine participants (janitors, *n* = 11; maintenance staff, *n* = 18) collected data about critical situations among older tenants residing in apartments in Sweden. Modifying the Critical Incident Technique (CIT) and applying a mixed-methods design, quantitative and qualitative data were collected and analyzed with descriptive statistics and thematic analysis, integrated through narrative. We found that older tenants asked staff for help with daily tasks. The staff identified CI management dilemmas in meeting older tenants’ support needs while following the housing company’s regulations, maintaining professional responsibilities, respecting individual work attitudes and preferences, and experienced a lack of competencies in some situations. Staff members were responsive to offering support in simple, practical, and emotional situations and in addressing matters they perceived as deficits in social and health services.

**What this paper adds**

• Knowledge about support needs among older tenants as experienced by public housing staff.
• Organizational dilemmas influence staff when supporting older tenants. The more complex the problem, the more the staff focused on following their formal work responsibilities to maintain impartiality, neutrality, and professionalism.
• Staff can provide familiarity and confidence to older tenants lacking informal networks or significant others and feel a sense of responsibility to prioritize helping those older tenants.

**Applications of study findings**

• This small-sized study paves the way for further exploration of factors related to aging in public housing, thus nurturing the development of support systems in local communities.
• The role of public housing staff in providing community support for older tenants should be thoroughly analyzed and planned as older tenants’ heterogeneous support needs exceed the staff’s professional competencies, training, and formal work responsibilities.
• Future research on these matters should explore public housing in municipalities of various sizes and include national surveys to guide the development of aging and housing policies.

## Introduction

With the aging of many Western populations, the need for supportive housing is increasing. In the United States of America (USA), 36% of households rent their homes, including apartments and single-family homes (Joint Center for Housing Studies, 2022). In Sweden, 29% of households rent apartments provided by Municipal Housing Companies [MHC] that are obligated to benefit people from all social groups regardless of income and background (Lindbergh & Wilson, 2016).

In recent decades, Swedish housing policies have changed the MHC objectives to become increasingly market- and efficiency-oriented (Grander, 2017). As regulated in the Public Municipal Housing Companies Act (Government Offices in Sweden, 2010), MHCs are run following business-like principles and include Corporate Social Responsibility (CSR) in their business model, thus providing a social benefit to the population (Blomé, 2012; Lindbergh & Wilson, 2016; Government Offices in Sweden, 2010).

Traditionally, janitors’ and maintenance staffs’ work in public housing companies involves maintaining the housing stock and common outdoor areas. Increasingly, these categories of staff attend courses in eviction prevention, integration projects, and conflict solving (Grander, 2015a, 2015b). However, janitors and maintenance staff moving away from a traditional service role involves dilemmas. Research shows that CSR initiatives across Swedish municipalities differ, and their effects can be categorized into three levels (Grander, 2015a). The first level involves the relationship between tenants and housing managers and the services provided. The second level involves social and cultural events. The third implies a moral and political obligation to benefit all households regardless of socioeconomic background.

According to the World Health Organization (WHO) (2007), public housing environments should consider older adults’ functional capacities. In their study, Zamora et al. (2019) included the perspectives of very old adults living in an age-friendly community in Canada. They found that the formal and informal design and structure of community support and health services improved the use of compensatory mechanisms contributing to aging in place (AIP). Kaplan et al. (2015) found that formal and informal support from health professionals and family was required to support AIP for older adults with neurocognitive disorders. Informal caregivers provided 70% of care and support, and relocation to a skilled care facility was hard to prevent. Bengtsson-Tops and Hansson’s (2014) study of landlords in Sweden and their experiences of housing tenants with severe mental illness identified mismanagement of apartments, disruptive behavior, and tension between neighbors. The landlords’ role in solving tenants’ housing problems involved ethical dilemmas, preconceptions, attitudes, and emotions that went beyond their professional obligations. However, to what extent such critical situations apply to janitors and maintenance staff regarding older tenants is unknown.

Public housing janitors and maintenance staff likely become involved when accidents have occurred in tenants’ homes. Swedish and international reports on home and leisure accidents show higher risks of falls among older adults (Callaghan, 2021; Ekberg, 2019). Lack of support and care after an accident can cause lasting damage and fatalities. Moreover, Swedish (Karlstedt, 2015) and Australian (Johnstone et al., 2020) studies found that home healthcare reforms have reduced support services for older adults. When working where older adults live, janitors and maintenance staff may be the first to identify health and social problems. Their experiences could bring older adults’ formal and informal support needs to light. Swedish MHC directives suggest that janitors and maintenance staff provide social support services, but there is a lack of knowledge about their professional responsibility and potential dilemmas in conducting and coordinating CSR tasks.

This study aimed to explore critical situations involving older tenants encountered by staff in a MHC in Sweden, what helped or hindered their work performance and outcomes, and their approaches and strategies to manage critical situations.

## Methods

### Study Design

**Figure 1.**
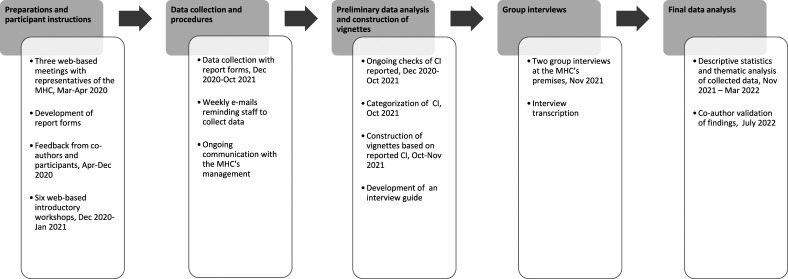
Overview of the data collection and data analysis process.

This explorative study had a sequential explanatory mixed-methods design (Fetters et al., 2013). For an overview of the data collection and analysis process, see Figure 1.

### Study Context

The study was conducted in collaboration with representatives of a MHC with 56 employees and a stock of approximately 2,300 apartments in a medium-sized municipality in the south of Sweden. The management had indicated that servicing older tenants with illness or disabilities sometimes caused problems for the janitors and maintenance staff in their day-to-day work. The janitors were primarily responsible for small repairs in the tenants’ apartments and buildings, including inspections before tenants moved out. The maintenance staff were responsible for sanitation in indoor and outdoor common areas.

### Participants

Thirty-one MHC staff members were approached to participate. Twenty-nine janitors (*n* = 11) and maintenance staff (*n* = 18), from now on referred to as staff, agreed to participate. For participant characteristics, see Table 1.

**Table 1.** Staff Characteristics, *N* = 29.

Characteristic	Janitors (*n* = 11)		Maintenance personnel (*n* = 18)	
Age, mean (SD)	41	(9)	48	(12)
Sex, *n* (%)				
Men	11	(100)	12	(66)
Women	0	(0)	6	(33)
Years employed in the company, mean (SD)	16	(8)	13	(9)

### Data Collection and Data Analysis Procedures

Applying a sequential explanatory mixed-methods design (Fetters et al., 2013), quantitative and qualitative data were collected. First, we used report forms for data collection with a modified version of the Critical Incident Technique (CIT). The CIT was developed to collect data during direct observations of practical handling processes. The method is suitable for analyzing and evaluating work processes and gaining expertise by registration of supporting and hindering factors critical to the targeted practice (Flanagan, 1954). Secondly, we conducted group interviews to increase the study’s empirical base, see Figure 1.

#### Co-Constructing Report Forms and Introductory Workshops With Staff

Guided by the staff’s work expertise (Flanagan, 1954), two digital report forms for data collection were developed by authors AY and MK in collaboration with managers and five staff members, see Figure 1. The first form was constructed to collect data on staff characteristics and was used once. The second form was developed to collect data on types of CI, including staff experiences and descriptions of where, when, and who was involved. Involving all co-authors, the report forms were optimized and tested in an iterative process.

The staff were introduced to the project and data collection procedures during six online workshops, each including four to six staff members. The digital report forms were tested using their digital devices, and no further adjustments were necessary to establish the final versions.

#### Data Collection

The staff collected data using report forms on the Sunet Survey platform. In total, 32 CI were registered, of which 23 were included in the study, see Table 2. Nine CI reports were excluded because they were either out of scope or had been handed over to a manager because the CI exceeded the staff’s work responsibilities.

**Table 2.** Description of Type of Critical Incidents (CI), When and Where they Happened, if Additional People were Involved, and Staff Satisfaction with Management, *N* = 23.

Critical Incident	CI No.	When		Location			Additional people Involved	Satisfactorily Managed		
		During regular working hours	During evenings, nights, or weekends	In the apartment	Common indoor area	Common outdoor area		Yes	No	Partly
Needing help to start car^c^	1	X	–	–	–	X	–	–	–	X
Fall accident in laundry room^f^	2	X	–	–	X	–	MHC staff	X	–	–
Needing help to empty attic and dispose waste^c^	3	X	–	–	X	–	MHC staff	X	–	–
Needing help to move furniture from attic to apartment^c^	4	X	–	X	X	–	–	X	–	–
Needing help to get the TV started^c^	5	X	–	X	–	–	–	X	–	–
Needing help to carry heavy rug^c^	6	X	–	–	X	–	–	X	–	–
Complaints about a noisy neighbor^e^	7	X	–	–	X	–	–	X	–	–
Not being satisfied with maintenance work in common area^d^	8	X	–	–	–	X	–	X	–	–
Convinced people entering the apartment at night^f^	9	X	–	X	–	–	MHC staff	–	X	–
Tenant getting lost, unable to find the way home^f^	10	X	–	–	–	X	Another tenant	X	–	–
Emptying apartment after tenant, so the housing company can hand it over to a new tenant^c^	11	X	–	X	–	–	Family member Another tenant MHC staff	–	–	X
Accusing neighbor of stalking^e^	12	X	–	–	–	X	Another tenant	X	–	–
Neighbors accusing each other of harassment^e^	13	X	–	–	–	X	Another tenant MHC staff	–	–	X
Neighbor storing items on another neighbor’s terrace^e^	14	X	–	–	–	X	Another tenant MHC staff	–	–	X
Fall accident during home repairs^d^	15	X	–	X	–	–	Family member	X	–	–
Need help to get out of the building when the elevator is out of order^d^	16	X	–	–	X	–	MHC staff	–	–	X
Call from a tenant about a tragic event in the family^g^	17	X	–	X	–	–	MHC staff	X	–	–
Neighbor accusing neighbor of harassment^e^	18	X	–	–	–	X	Family member Another tenant MHC staff Staff from another authority	–	–	X
Neighbor accusing neighbor of harassment^a,e^	19	X	–	–	–	X	Another tenant MHC staff	–	X	–
Attentive tenant worried about neighbor not being seen for days^f^	20	X	–	X	–	–	Another tenant MHC staff Blue light personnel	X	–	–
Breach of rules of conduct, mismanagement, and odor from tenant’s apartment^f^	21	X	–	X	–	–	Family member Another tenant MHC staff Staff from another authority	X	–	–
Breach of rules of conduct, mismanagement, and odor from a tenant’s apartment^b,f^	22	X	–	X	–	–	Family member MHC staff Staff from another authority	X	–	–
Lack of personal hygiene^f^	23	X	–	–	–	X	MHC staff Staff from another authority	–	X	–

^a^ = Continuation of CI 18.

^b^ = Continuation of CI 21.

CI categorized in vignettes:

^c^ = a simple quick task 1, 3–6, 11.

^d^ = repairs and maintenance 8, 15–16.

^e^ = neighbors 7, 12–14, 18–19.

^f^ = illness 2, 9–10, 20–23.

^g^ = loneliness 17.

#### Preliminary Data Analysis and Construction of Vignettes

**Figure 2.**
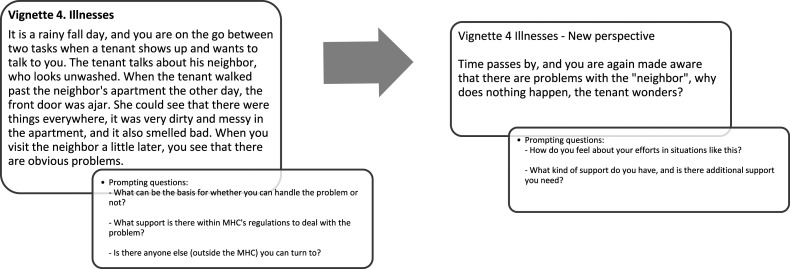
Example of a vignette used in the group interviews.

Data from the digital report forms were grouped based on the type of CI, resulting in five vignettes (Alok, 2019). The vignettes were developed to prompt additional qualitative data collection to further explore the staff’s management, approaches, and strategies regarding CI events. The vignettes illustrated CI events such as (1) a quick and simple task, (2) repairs and maintenance, (3) neighbors, (4) illness, and (5) loneliness phrased to facilitate discussion from staff regardless of whether they had reported a CI. To illustrate the vignettes’ content and design, Figure 2 presents one example of the most frequent CI reported (illness).

#### Group Interviews

AY and MK conducted two group interviews, using the vignettes as a topic-focused interview guide (see Figure 2) at the MHC premises. Four staff members had left their job at the time of the group interviews, and four could not participate; thus, 21 staff members participated, divided into two groups (*n* = 10; *n* = 11). MK facilitated and structured the group interviews, while AY observed and took detailed notes, including quotes. Each group interview lasted two hours and was audio-recorded.

#### Final Data Analysis

Using the statistical analysis software IBM SPSS 27, quantitative data on staff characteristics were analyzed descriptively. Quantitative CI reports were compiled and consecutively presented, including context, time, place, involvement of others, and management quality, see Table 2. Qualitative data were analyzed using the thematic analysis approach by Braun and Clarke (2019). Using this approach, data is reflexively interpreted to identify patterns and create themes and subthemes. Data from digital reports and interviews were integrated through narrative by weaving quantitative and qualitative findings together theme by theme (Fetters et al., 2013).

**Figure 3.**
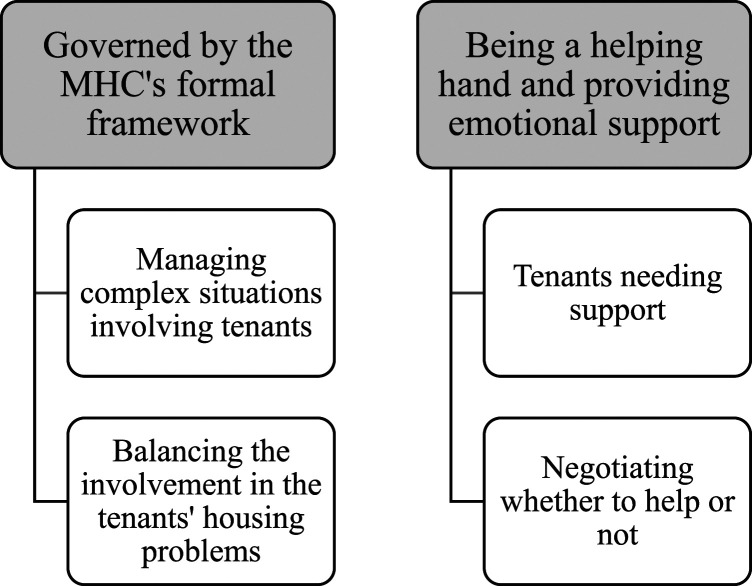
Overview of themes and subthemes presented as Findings. Note: MHC = (Municipal Housing Company).

First, authors AY and MK independently listened to audio recordings and read the field notes to familiarize themselves with the data. We identified preliminary themes of (1) prioritizing time and planning tasks, (2) work safety and responsibilities, (3) organizational dilemmas, complex tasks, knowledge and involvement reluctance, and (4) emotional support. To improve trustworthiness, AY and MK applied an ongoing collaborative, reflective approach to discussing developing themes. This iterative process included the identification of nuances, similarities, and differences in the data and patterns in explanations and descriptions provided by participants. Subsequently, two themes and four subthemes were developed, see Figure 3. Having access to the qualitative and quantitative data for validity checks, co-authors MG, AMF, and SI critically reviewed the findings, followed by discussions involving all authors and final refinement.

## Findings

Eight janitors reported 21 of the 23 CI. All CI took place during regular working hours, and 39% took place in common outdoor areas, 35% in older tenants’ apartments, and 26% in common indoor areas. In 52% of the CI, the staff were contacted by an older tenant. In 18%, the staff themselves discovered the CI. For the remaining 31%, another tenant or someone outside the organization contacted the staff. The staff thought 61% of the CI were solved satisfactorily, see Table 2.

The analysis generated two themes, each with two subthemes, see Figure 3. The first theme illustrates the staff being “governed by the MHC formal regulations.” The second illustrates the staff “being a helping hand and providing emotional support.” Throughout the text, CI numbers refer to Table 2.

### Governed by the MHC’s Formal Regulations

This theme illustrates how the MHC’s formal regulations only partly guided the staff’s practical work. The organization of the work and staff’s competencies influenced their involvement when servicing tenants, causing uncertainty and dilemmas in subjectively judging the right or wrong thing to do.

#### Managing Complex Situations Involving Tenants

Tenants not following the MHC regulations, placing items or garbage on the stairways or another tenant’s terrace (CI 14) were CI occurring in common areas. According to the staff, managing such CI could involve bringing neighbors together to discuss respecting one another’s spaces. In recurring disputes, the staff handed such cases over to the manager since further involvement exceeded their responsibilities; 33% of tenant disputes were considered managed satisfactorily. Tenant disputes highlighted difficulties among tenants in terms of habits and housing values. From experience, the staff knew that tenant disputes were hard to solve within the MHC regulations. They expressed powerlessness:

“We do not feel there is much we can do (…) some tenant disputes are impossible to solve and can last several years.”

One-third of the CI involved tenants with symptoms potentially related to cognitive difficulties. Those CI were handed over to the manager, who followed up on them by contacting the social or home healthcare services to bring awareness to the problem. Occasionally such contacts had helped, but problems could reoccur if the tenants’ collaboration with the social services failed if they forgot to take their medication (CI 23) or misused alcohol. Some of those tenants were unable to seek help and risked eviction. According to the staff, collaboration with social services was challenging because social services often appeared to take the side of older tenants to protect them from eviction. Still, 71% of the staff expressed satisfactory outcomes in such CI, including handing them over to the manager because they were untrained, and such cases exceeded their responsibilities. Allowing older tenants to disrupt the premises and daily lives of their neighbors was proof of society’s weakness in meeting the needs of vulnerable older tenants:

“Aging in place policies and limited supply of purpose-built housing have led to unacceptable neglect of vulnerable citizens, considering we live in a rich welfare country like Sweden.”

Some tenants appearing to have cognitive difficulties approached staff because they heard sounds or people talking at night, making them scared and unable to sleep well (CI 7, 9). Occasionally, those tenants got lost in the housing area (CI 10). Unable to calm the tenants, the staff would consider involving older tenants’ family members, which was the case in 22% of the CI reported. Sometimes, there was no family or significant other to contact, leaving the staff concerned about the older tenants’ well-being in doubt about how to solve the situation responsibly.

Other examples of complex situations were CI related to neighbors shouting and knocking on walls and doors (CI 7) or stalking, threatening, or harassing neighbors (CI 12, 13, 18, 19). Sometimes tenants would involve their family members, who could also pressure the staff for help. In other CI, an unpleasant odor from a neighbor’s apartment and a tenant’s lack of personal or home hygiene was reported (CI 21–23). Such CI could be ongoing, recurring, and known to the staff for a long time, which was the case in 17% of the CI reported. Staff said those tenants could pose a health and safety risk to themselves and their neighbors. Smells from animals were easy to confirm; however, neglect of an apartment was hard to identify unless inspected. Having conversations with older tenants about apartment maintenance was challenging due to the sensitive nature of the topic:

“Perhaps a little joke said with a wink works better than a direct statement about the apartment needing cleaning.”

#### Balancing the Involvement in the Tenants’ Housing Problems

Regarding older tenants’ complaints about aggressive neighbors, the staff were merely able to recommend calling the police, security companies, or MHC managers. Complaints could be filed anonymously, and the MHC could take future action by gathering repeated cases related to specific tenants. Lacking knowledge about the older tenants’ private interactions and conversations, the staff encouraged the older tenants to solve disagreements among themselves, balancing their involvement to work formally, professionally, and impartially:

“We must stay neutral. We must retain the trust of all the tenants because we can be called on to do work in their homes.”

The staff’s knowledge of the housing area and the tenants helped them manage emerging incidents. Some housing areas had similar problems, probably due to tenants living there for a long time, while others had a large turnover of residents and various challenges. Establishing relationships with tenants in the smaller areas was easier. In the larger areas, some older tenants lived secludedly. According to the staff, older tenants were more sensitive to neighbors and complained more than younger tenants. The staff explained how having neighbors caused some older tenants anger, arguments, and grief. Some had moved to other apartments in the housing area.

“Some tenants just don’t get along and don’t like each other due to behaviors or habits.”

### Being a Helping Hand and Providing Emotional Support

This theme illustrates the staff’s approach to older tenants asking for practical help and emotional support. The MHC values included making the housing area a good and safe place to live and valuing the tenants. Such matters often clashed with their formal work responsibilities, reflected in dilemmas for the staff to meet the support needs of older tenants they knew lacked formal and informal networks. Sometimes older tenants’ needs prompted the staff to take safety risks.

#### Tenants Needing Support

Twenty-six % of the CI reported involved practical needs like helping to move furniture between an apartment and storage and driving it to the local tip (CI 3, 4), tuning a TV signal (CI 5), helping to start a car (CI 1), emptying and cleaning an apartment (CI 11), providing personal assistance and carrying a powered wheelchair up/down some steps (CI 16). Such CI was fairly easy to solve, requiring no follow-up and predominately satisfactorily managed.

Although only one CI concerning loneliness was reported, staff working in the common outdoor area said lonely older tenants approached them daily to chat. The older tenants knew the staff’s work routines and when and where they could meet them. Due to such conversations, the staff had some insight into some older tenants’ personal situations. Some staff perceived it as a sign that they were good listeners and competent in providing emotional support. Generally, the staff expressed satisfaction with these conversations and said they were a social part of their work and provided feelings of safety in the housing area.

Staff exemplified they provided emotional support to an older tenant who had experienced a serious incident in the family and not having anyone else to talk to (CI 17). In another accident related to a major housing renovation, staff provided emotional support to an older tenant’s spouse (CI 15). Expressing their concern about lonely older tenants, some staff were observant of those not seen on usual rounds:

“The really lonely tenants fly under the radar; however, they do exist.”

To show their concern, staff sometimes asked an older tenant whether they had seen their neighbor. They said that neighbors paid less attention to each other nowadays. However, due to a neighbor’s attentiveness, staff discovered that an older tenant had an accident in their apartment and was unable to call for help (CI 20). The staff would also encourage older tenants and assist them in participating in a cafe project to reduce loneliness among older tenants.

#### Negotiating Whether to Help or Not

The staff’s willingness to help older tenants manage tasks outside their formal responsibilities varied depending on the task, available time, individual preferences, and work safety. Most staff were flexible and wanted to help. In approximately 70% of the CI, others than the reporting staff (predominantly a colleague) were involved in the CI management. Such CI was reported inside apartments and in indoor and outdoor common areas. Helping with practical tasks like moving heavy furniture (CI 3, 4), helping an older tenant who had fallen up from the floor (CI 2), or carrying an older tenant and his powered wheelchair (CI 16) were not part of the staff’s formal work responsibilities, causing dilemmas as to whether to help. This was reflected in staff saying that if they reacted instinctively and undertook such tasks, it caused dilemmas between helping and upholding safety guidelines and thereby taking safety risks:

“A colleague was injured when he helped a tenant who had fallen on the street. The [company´s] work insurance did not cover his injury because it was not part of his job to lift anybody in such a situation.”

According to the staff, they performed worthwhile services and said that older tenants expressed satisfaction with the support received. However, they wanted to be appreciated for their help and tried to avoid giving the impression that they were available at the snap of the older tenants’ fingers. If the staff knew an older tenant had family members or significant others, they asked them to be involved in helping out. If staff did not have time right away, they arranged another time to help. In this way, they prioritized and showed a willingness to help older tenants they knew lacked informal networks:

“We know some tenants have small social networks, and we more often help those tenants.”

Scheduled and unexpected elevator disruptions, causing problems for those older tenants dependant on an elevator to leave home, were usual problems. Even though older tenants could contact providers offering transport services, or the SOS alarm number, to help them up/down the stairs with heavy equipment, such as powered wheelchairs, these providers prioritized acute emergencies more than non-acute personal assistance. Therefore, staff were called to assist and carry an older adult’s powered wheelchair up/down some stairs to the first floor several times a week while waiting for elevator repair (CI 16). The staff debated their obligations to help. Some would not take responsibility for carrying persons or expensive equipment due to safety issues, strictly following the formal guidelines. Some would only help in situations they considered important, such as a doctor’s appointment, while others faced the dilemma without hesitating to help despite the risk involved:

“Sometimes there is no common sense. When you meet a tenant asking for help, you just do it.”

## Discussion

To our knowledge, this study is the first to focus on housing staff supporting older tenants in public housing. CI management dilemmas were identified between meeting older tenants’ support needs while following formal MHC regulations, staff competencies and formal responsibilities, and their individual preferences and work attitudes. Although the MHC’s values included making the housing area a good and safe place to live and valuing the tenants, the staff were approached for support by older tenants having challenges coping with practical, social, and emotional problems belonging to their private sphere. Staff felt a sense of responsibility and powerlessness when older tenants’ support needs were unmet. Although staff in Sweden attend regular courses to increase their CSR skills, this study highlights dilemmas in managing the different CI identified.

We found that CI among older tenants with cognitive difficulties, including home and hygiene challenges, were most commonly reported. The findings deserve attention as the staff involved were untrained in recognizing illness and had difficulties supporting those older tenants. In Sweden, municipalities are responsible for providing social and home care services; however, older tenants can refuse to accept offered support (National Board of Health and Welfare, 2012). Governed by the MHC formal regulations, we found that staff managed complex situations by balancing their involvement. Handing over the CI to their manager was an appropriate management strategy to ensure quality, competency, and safety. In recurring CI, despite the municipality’s involvement, staff would question the municipal services provided, AIP policies, and the Swedish welfare system’s general ability to support vulnerable older tenants. According to Iecovich (2014), improving interdisciplinary teamwork, financing, and reducing legal problems can improve coordination and collaboration between municipal partners. Our findings are similar to Bengtsson-Tops and Hansson’s qualitative study (2014) of Swedish landlords and their experiences with tenants with mental illness whose social and health needs were difficult for the housing company to support. Their study concluded that tenants’ support needs exceed the landlords’ professional and formal work obligations. However, as in our study, landlords created awareness among housing managers and municipality partners and therefore played an important role in identifying, addressing, and preventing risks to health and well-being. Likely, support needs among older tenants would be unidentified and not attended to without public housing staff’s engagement.

Our findings show that CI management occurred in direct day-to-day encounters between older tenants and the staff. Mostly, older tenants living alone, lacking informal networks or significant others, approached the staff for necessary support. According to Iecovich (2014), informal networks usually provide support belonging to the private sphere. To the staff in our study, supporting older tenants was an extra workload, which the individual staff members negotiated and related to time available and their willingness to assist. Grander’s research (2015a) on CSR in MHC in Sweden found that staff generally feel they contribute positively to providing support services to tenants. We found such support included simple, practical tasks, emotional support, or conversations that appeared to be an important social part of staff work they felt competent with. This observation is supported by the finding indicating that, for some older tenants, the staff became familiar and trusted individuals they felt comfortable approaching. In complex CI, supporting older tenants involved dilemmas where staff felt that the best solution was to limit their involvement and stay neutral to retain the trust of all tenants. Although the MHC had a department for managing tenant conflicts, persistent conflicts led to staff feeling powerless in the face of unrealistic expectations from older tenants and their families of the staff’s role in resolving them. Our findings indicate that the professionals in the MHC managing tenant conflicts protected some older tenants from housing eviction. Possible reasons may be a lack of easy access to alternative housing and formal support systems or cuts in home healthcare and social services (Iecovich, 2014; Karlstedt et al., 2015). Our findings suggest opportunities for MHC to enable social involvement and tenant participation to make environments friendlier to older adults. Promoting networking among tenants is also a way to create informal, supportive systems (Iecovich, 2014; WHO, 2007) and to approach those older tenants who are reluctant to reach out to established public service authorities. Furthermore, increased access to purpose-built facilities for older tenants with difficulties due to illness or functional decline should be considered because AIP is not secure or desirable for all.

We found that CI management involving practical tasks, for example, heavy lifting, could lead to staff not complying with their work safety guidelines, but older tenants’ needs could appeal to some staff’s sense of care and responsibility. In emergency elevator disruptions, it may be argued that the staff would carry out heavy lifting to ensure older tenants’ autonomy and independence due to their sense of responsibility. Most research on janitors’ work shows that their work environment is associated with physical wear, stress, and burnout (Charles et al., 2009; Estacio, 2021; Green et al., 2019; Schwartz et al., 2019). We found that the staff considered the best option in each CI. The shortcoming of work insurance coverage identified in our study can guide future CSR requirements in MHC and the ongoing training and appropriate technical equipment needed for future improvements in CI management.

According to Grander (2015a), providing staff with the necessary CSR qualifications is the responsibility of the MHC management. Salonen’s (2015) research on MHC in Sweden states that CSR initiatives should be thoroughly analyzed to benefit the residing tenants and the MHC socially and financially. Staugård (2011) states that professionals are provided with formally learned skills, securing quality for specific work practices, thereby reducing dilemmas and uncertainty in work processes. Despite MHC staff attending basic courses in social skills (Grander, 2015a), they may lack the necessary competencies to professionally support the heterogeneous support needs of older tenants’ AIP. Our findings suggest that public housing staff are responsive to older tenants’ simple, practical, and emotional support needs and involved in addressing matters they feel are overlooked by municipal social and health services. This study provided insight into the staff’s work and competency dilemmas concerning older tenants’ housing situations. Such perspectives should be included in organizational decision-making based on their work experiences among older tenants.

### Strengths and Limitations

This project was initiated in March 2020, just as the COVID-19 pandemic started. This contributed to postponing and extending the data collection period, thus influencing the researchers’ contact with the participating staff and minimizing their direct contact with older tenants because of national restrictions. In combination with the staff’s lack of experience in participation in research, this resulted in findings based on only 23 CI reports despite weekly reminders via e-mail and repeated efforts to make progress in the data collection. To increase the empirical base for the study, we adapted the study design by conducting two group interviews to expand on specific CI experiences. Data verification was possible using vignettes in the group interviews, and this approach was instrumental in capturing the depth and breadth of the CI registered in the digital report forms.

Although the character of CI observed in this study appears universal based on existing knowledge about challenges older adults with compromised health may face in their everyday lives, to our knowledge, this study is the first of its kind. Only after similar studies in other countries can the global universality of the findings be discussed in a valid manner. The findings should be interpreted with this in mind.

## Conclusion

This small-sized study gives insight into hitherto unknown perspectives of staff working in a MHC in Sweden and their encounters with older tenants. The findings shed light on organizational issues influencing staff roles based on qualitative and quantitative data. Dilemmas appeared between meeting older tenants’ support needs and following the MHC governing regulations, professional responsibilities and competencies, and staff preferences and work attitudes. Managing simple and complex CI, the staff balanced their involvement in critical situations related to tenants’ illnesses, neighbor disputes, practical help, and emotional support. The findings showcase organizational and professional issues that influence MHC staff’s ability to judge and manage older tenants’ support needs confidently. Politicians, stakeholders, and public housing providers should recognize that housing staff juggle competing roles and work dilemmas. Housing staff perspectives should be included in organizational decision-making, grounded in their work experiences among older tenants. While more research on such matters is warranted, the findings have potential to guide careful reassessments of the staff categories and training needed to support older tenants while maintaining high-quality services and safety in public housing.

## Supplemental Material

Supplemental Material - Support Needs Among Older Tenants Living in Public Housing in Sweden: Perspectives of Janitors and Maintenance Staff Click here for additional data file. Supplemental Material for Support Needs Among Older Tenants Living in Public Housing in Sweden: Perspectives of Janitors and Maintenance Staff by Agata Yadav, Marianne Kylberg, Marianne Granbom, Agneta Malmgren Fänge, and Susanne Iwarsson in Journal of Applied Gerontology.

## References

[bibr1-07334648231169130] Alok S. (2019). Vignette methodology: An illustration from conflict research. In Gupta M. Shaheen M. Reddy K. (Eds.), Qualitative techniques for workplace data analysis (pp. 117–143). IGI Global. 10.4018/978-1-5225-5366-3.ch006

[bibr2-07334648231169130] Bengtsson-Tops A. Hansson L. (2014). Landlords’ experiences of housing tenants suffering from severe mental illness: A Swedish empirical study. Community Mental Health Journal, 50(1), 111–119. 10.1007/s10597-013-9596-4 23361470

[bibr3-07334648231169130] Blomé G. (2012). Corporate social responsibility in housing management: Is it profitable? Property Management, 30(4), 351–361. 10.1108/02637471211249498

[bibr4-07334648231169130] Braun V. Clarke V. (2019). Reflecting on reflexive thematic analysis. Qualitative Research in Sport, Exercise and Health, 11(4), 589–597. 10.1080/2159676X.2019.1628806

[bibr5-07334648231169130] Callaghan M. (2021, June 24). A literature review of factors which cause and mitigate against injury in the home. Scottish Community Safety Network. https://www.safercommunitiesscotland.org/wp-content/uploads/Causation-Factors-Unintentional-Harm-in-Home-Final-Report-SCSN.pdf

[bibr6-07334648231169130] Charles L. Loomis D. Demissie Z. (2009). Occupational hazards experienced by cleaning workers and janitors: A review of the epidemiologic literature. Work, 34(1), 105–116. 10.3233/WOR-2009-0907 19923681

[bibr7-07334648231169130] Ekberg J. (2019, January 22). Strengthened work against home and leisure accidents. The Swedish Civil Contingencies Agency. https://www.msb.se/siteassets/dokument/om-msb/vart-uppdrag/regeringsuppdrag/2019/starkt-arbete-mot-hem-och-fritidsolyckor-2019.pdf

[bibr8-07334648231169130] Estacio D. (2021). Janitor’s attitudes: Their effect on performance at La consolacion university phippines SY 2020-2021. International Journal of Multidisciplinary: Applied Business and Education Research, 2(8), 664–676. 10.11594/ijmaber.02.08.05

[bibr9-07334648231169130] Fetters M. D. Curry L. A. Creswell J. W. (2013). Achieving integration in mixed methods designs-principles and practices. Health Services Research, 48(6 Pt 2), 2134–2156. 10.1111/1475-6773.12117 24279835 PMC4097839

[bibr10-07334648231169130] Flanagan J. (1954). The critical incident technique. Psychological Bulletin, 51(4), 327–358. 10.1037/h0061470 13177800

[bibr11-07334648231169130] Government Offices in Sweden . (2010, June 3). Act (2010:879) on non-profit municipal housing stock companies. Government Offices in Sweden. https://rkrattsbaser.gov.se/sfst?bet=2010:879

[bibr12-07334648231169130] Grander M. (2015a). Social responsibility of the municipal housing companies. In: Salonen T. (Ed.), The benefit of municipal housing companies (pp. 160–188). Liber.

[bibr13-07334648231169130] Grander M. (2015b). Social responsibility on business terms - interpretations and consequences of the Act on municipal housing stock companies. In: Salonen T. (Ed.), The benefit of municipal housing companies (pp. 189–210). Liber.

[bibr14-07334648231169130] Grander M. (2017). New public housing: A selective model disguised as universal? Implications of the market adaptation of Swedish public housing. International Journal of Housing Policy, 17(3), 335–352. 10.1080/19491247.2016.1265266

[bibr15-07334648231169130] Green D. R. Gerberich S. G. Kim H. Ryan A. D. McGovern P. M. Church T. R. Schwartz A. Arauz R. F. (2019). Janitor workload and occupational injuries. American Journal of Industrial Medicine, 62(3), 222–232. 10.1002/ajim.22940 30675912

[bibr16-07334648231169130] Iecovich E. (2014). Aging in place: From theory to practice. Anthropological Notebooks, 20(1), 21–33. https://www.researchgate.net/publication/288116698_Aging_in_place_From_theoy_to_practice

[bibr17-07334648231169130] Johnstone G. Dickins M. Lowthian J. Renehan E. Enticott J. Mortimer D. Ogrin R. (2021). Interventions to improve the health and wellbeing of older people living alone: A mixed-methods systematic review of effectiveness and accessibility. Ageing and Society, 41(7), 1587–1636. 10.1017/S0144686X19001818

[bibr18-07334648231169130] Joint Center for Housing Studies . (2022, January 12). America’s rental housing 2022. Joint Center for Housing Studies of Harvard University. https://www.jchs.harvard.edu/sites/default/files/reports/files/Harvard_JCHS_Americas_Rental_Housing_2022.pdf

[bibr19-07334648231169130] Kaplan D. B. Andersen T. C. Lehning A. J. Perry T. E. (2015). Aging in place vs. relocation for older adults with neurocognitive disorder: Applications of Wiseman’s behavioral model. Journal of Gerontological Social Work, 58(5), 521–538. 10.1080/01634372.2015.1052175 26016530 PMC4506221

[bibr20-07334648231169130] Karlstedt M. Wadensten B. Fagerberg I. Pöder U. (2015). Is the competence of Swedish registered nurses working in municipal care of older people merely a question of age and postgraduate education? Scandinavian Journal of Caring Sciences, 29(2), 307–316. 10.1111/scs.12164 25213399

[bibr21-07334648231169130] Lindbergh L. Wilson T. (2016). Strategic management in Swedish municipal housing. Property Management, 34(2), 136–157. 10.1108/PM-07-2015-0030

[bibr22-07334648231169130] National Board of Health and Welfare . (2012, February 14). The value base in the social services’ care for the elderly. National Board of Health and Welfare. https://www.socialstyrelsen.se/globalassets/sharepoint-dokument/artikelkatalog/foreskrifter-och-allmanna-rad/2012-2-20.pdf

[bibr23-07334648231169130] Salonen T. (2015). Social responsibility of the municipal housing companies. In: Salonen T. (Ed.), The benefit of municipal housing companies (pp. 1–24). Liber.

[bibr24-07334648231169130] Schwartz A. Gerberich S. Albin T. Kim H. Ryan A. D. Church T. R. Green D. R. McGovern P. M. Erdman A. G. Arauz R. F. 2020). The association between janitor physical workload, mental workload, and stress: The SWEEP study. Work, 65(4), 837–846. 10.3233/WOR-203135 32310213

[bibr25-07334648231169130] Staugård H. (2011). The concept of professions. In: Johansen M. B. Olesen S. G. , (Eds), The sociology and knowledge base of professions (pp.161–175). Systime.

[bibr26-07334648231169130] World Health Organization [WHO] . (2007, October 10). Global age-friendly cities: A guide. World Health Organization. https://apps.who.int/iris/handle/10665/43755

[bibr27-07334648231169130] Zamora F. M. V. Kloseck M. Fitzsimmons D. A. Zecevic A. Fleming P. (2019). Use of community support and health services in an age-friendly city: The lived experiences of the oldest-old. Cities & Health, 4(1), 107–116. 10.1080/23748834.2019.1606873

