# Supplementary material for: Support Needs Among Older Tenants Living in Public Housing in Sweden: Perspectives of Janitors and Maintenance Staff
Source: J Appl Gerontol. 2023 Apr 25;42(8):1727–37. doi: 10.1177/07334648231169130 (PMC10394964; doi:10.1177/07334648231169130)
Supplement: Supplemental Material - Support Needs Among Older Tenants Living in Public Housing in Sweden: Perspectives of Janitors and Maintenance Staff [file sj-pdf-1-jag-10.1177_07334648231169130.pdf]

COREQ (COnsolidated criteria for REporting Qualitative research) Checklist

A checklist of items that should be included in reports of qualitative research

Topic	Item No.	Guide Questions/ Description	Author Responses
**Domain 1: Research team and reflexivity**			
Personal Characteristics			
Interviewer/facilitator	1	Which author/s conducted the interview or focus group?	Marianne Kylberg (MK): Interviewer<br>Agata Yadav (AY): Observer, taking notes.
Credentials	2	What were the researcher's credentials? E.g. PhD, MD	MK: Ph.D. and Lecturer<br>AY: Ph.D. Student.<br>Marianne Granbom (MG): Ph.D. Senior lecturer and associate professor.<br>Agneta Malmgren Fänge (AMF): Ph.D. and senior lecturer.<br>Susanne Iwarsson (SI): Professor.
Occupation	3	What was their occupation at the time of the study?	MK: Lecturer<br>AY: Ph.D. student, Active and healthy ageing research group.<br>MG: Assistant senior lecturer. Active and healthy ageing research group.<br>AMF: Senior lecturer, head of Department of health sciences.<br>SI: Head of Active and healthy ageing research group.
Gender	4	Was the researcher male or female?	Females.
Experience and training	5	What experience or training did the researcher have?	MK: Lecturer, reg. occupational therapist, trained for qualitative research during her Ph.D. education.<br>AY: Ph.D. student course: Applied Qualitative Methodology (1,5 hp)<br>MG: Assistant senior researcher, reg. occupational therapist.<br>AMF: Senior researcher, reg. occupational therapist.<br>SI: Professor. reg. occupational therapist.
Relationship with participants			
Relationship established	6	Was a relationship established prior to study commencement?	The Municipality-owned Housing Company (MHC) is a formalized partner in a Thematic Collaboration Initiative financed by Lund University.
Participant knowledge of the interviewer	7	What did the participants know about the researcher? e.g. personal goals, reasons for doing the research	The research staff involved in the focus groups were presented to the participants at the start of the project.
Interviewer characteristics	8	What characteristics were reported about the inter-	Included in the presentation of the interviewer, see above.

		viewer/facilitator? e.g. Bias, assumptions, reasons and interests in the research topic	
**Domain 2: Study design**			
Theoretical framework			
Methodological orientation and Theory	9	What methodological orientation was stated to underpin the study? e.g. grounded theory, discourse analysis, ethnography, phenomenology, content analysis	We used an explorative study with a sequential explanatory mixed-methods design.<br><br>We applied a modified version of the Critical Incident Technique (CIT) to collect data during direct observations.<br><br>Data was analysed using a thematic analysis approach and descriptive analysis, integrated through narrative by weaving quantitative and qualitative findings together, theme by theme.
Sampling	10	How were participants selected? e.g. purposive, convenience, consecutive, snowball	We collaborated with the MHC management, including all janitors and maintenance staff employed at the time of project start.
Method of approach	11	How were participants approached? e.g. face-to-face, telephone, mail, email	MHC staff were approached personally by their team leader.
Sample size	12	How many participants were in the study?	Twenty-nine.
Non-participation	13	How many people refused to participate or dropped out? Reasons?	Four staff members dropped out due to leaving their employment during the study period.
Setting			
Setting of data collection	14	Where was the data collected? e.g. home, clinic, workplace	At the workplace during work hours.
Presence of non-participants	15	Was anyone else present besides the participants and researchers?	During the registration of CIT, residents and in the MHC stock of apartments were present. During the group interviews only participants and researchers were present.
Description of sample	16	What are the important characteristics of the sample? e.g. demographic data, date	The staff's expertise from working in a housing area where older tenants reside, related to the study's aim.<br><br>Sex:<br>Men 11.<br>Woman: 18.<br><br>Mean age:<br>Janitors, 41 (n=11).<br>Maintenance staff, 48 (n=18).

			Mean years of employment in the company:<br>Janitors: 16.<br>Maintenance staff: 18.
Data collection			
Interview guide	17	Were questions, prompts, guides provided by the authors? Was it pilot tested?	All participants tested the CIT report forms during workshops, followed by revisions until the final format was established.<br><br>The interview guide was developed by the research team, but not pilot tested.
Repeat interviews	18	Were repeat interviews carried out? If yes, how many?	No, but after the group interviews the researchers asked participants questions about specific meanings of reported critical incidents, to validate survey data.
Audio/visual recording	19	Did the research use audio or visual recording to collect the data?	The group interviews were recorded.
Field notes	20	Were field notes made during and/or after the interview or focus group?	Yes, during and after the group interviews by AY, first author.
Duration	21	What was the duration of the interviews or focus group?	One hour for each group interview.
Data saturation	22	Was data saturation discussed?	Yes.
Transcripts returned	23	Were transcripts returned to participants for comment and/or corrections?	No.
**Domain 3: analysis and findings**			
Data analysis			
Number of data coders	24	How many data coders coded the data?	AY and MK; first and second authors.
Description of the coding tree	25	Did authors provide a description of the coding tree?	AY and MK, first and second authors developed, exchanged and reflected on a coding tree. All members in the research group discussed the themes in an iterative process.
Derivation of themes	26	Were themes identified in advance or derived from the data?	Derived from data.
Software	27	What software, if applicable, was used to manage the data?	No.
Participant checking	28	Did participants provide feedback on the findings?	Partly, with selected participants to validate CIT reports.
Reporting			

Quotations presented	29	Were participant quotations presented to illustrate the themes/findings?<br>Was each quotation identified? e.g. participant number	Yes.
Data and findings consistent	30	Was there consistency between the data presented and the findings?	Yes.
Clarity of major themes	31	Were major themes clearly presented in the findings?	Yes.
Clarity of minor themes	32	Is there a description of diverse cases or discussion of minor themes?	Yes.

Developed from: Tong A, Sainsbury P, Craig J. Consolidated criteria for reporting qualitative research (COREQ): a 32-item checklist for interviews and focus groups. *International Journal for Quality in Health Care*. 2007. Volume 19, Number 6: pp. 349 – 357

Additional Guidelines for Completing the COREQ Checklist for *Journal of Applied Gerontology*:

- This checklist will be published online as supplementary material and we require it to be in the form of a publishable table. Please make sure that material does not bleed outside of cells, etc.
- This checklist is designed to direct readers to relevant material in the manuscript. Where applicable, please direct readers to various sections of the manuscript, such as a Methods section, Conceptual Framework, table or figure. Pages may shift during the publication process so please avoid directing readers to specific page numbers.
- This checklist also is designed to supplement information that may not be reported in the text and/or provide additional details related to information that is reported in the text.

Once you have completed this checklist, please save a copy and upload an anonymized version of it as part of your *Journal of Applied Gerontology* submission. DO NOT include this checklist as part of the main manuscript document. It must be uploaded as a separate supplemental file. If the paper is accepted, a non-anonymized version should be provided with the final submission of the main manuscript.
